# Developmental Trends of Metabolic Syndrome in the Past Two Decades: A Narrative Review

**DOI:** 10.3390/jcm14072402

**Published:** 2025-03-31

**Authors:** Ahmad A. Obeidat, Mousa N. Ahmad, Mai A. Ghabashi, Awfa Y. Alazzeh, Salam M. Habib, Dalia Abu Al-Haijaa, Firas S. Azzeh

**Affiliations:** 1 Human Nutrition and Dietetics, Department of Nutrition and Food Science, The University of Jordan, Amman 11942, Jordan; mosnuman@ju.edu.jo (M.N.A.); s.habib@ju.edu.jo (S.M.H.); 2 Clinical Nutrition Department, Faculty of Applied Medical Sciences, Umm Al-Qura University, Makkah 21955, Saudi Arabia; 3 Department of Clinical Nutrition, Faculty of Applied Medical Sciences, University of Ha’il, Ha’il 2440, Saudi Arabia; 4 Department of Diet Therapy Technology and Dietetics, Faculty of Allied Medical Sciences, Zarqa University, Al-Zarqa 13110, Jordan; dabualhaijaa@zu.edu.jo

**Keywords:** metabolic syndrome, type 2 Diabetes mellitus, obesity, insulin resistance, waist circumference, cardiovascular disease

## Abstract

**Background/Objectives**: Metabolic syndrome (MetS) is a complex disorder characterized by insulin resistance (IR), central obesity, atherogenic dyslipidemia, and higher glucose levels. It increases the risk of cardiovascular disease (CVD) and type 2 diabetes mellitus (T2DM), imposing an economic burden on the healthcare system. However, the historical origins of MetS as well as the development and evolution of its definitions have not been conclusively documented in the literature. This study seeks to enhance the understanding of the developmental trends of MetS during the preceding two decades, placing particular emphasis on the definition, diagnosis and prevalence. **Methods**: An extensive search was performed from 1920 to 2023 across prominent scientific research engines, including Scopus, PubMed, MDPI, and others. **Results**: Despite advancements, many aspects of MetS remain inadequately understood. As the understanding of the nature and pathophysiology of MetS progresses, the development and refinement of its diagnostic criteria, and assessment and treatment guidelines will continue. Additionally, there exists significant variation in the global prevalence of metabolic syndrome, ranging from 14 to 39%. This prevalence is projected to increase due to the adoption of less healthy dietary patterns and sedentary lifestyles. The observed disparities in metabolic syndrome prevalence can be attributed to multiple factors, including demographic characteristics. Furthermore, the lack of a standardized definition across studies also contributes to the variation in reported prevalence rates. **Conclusions**: Further studies focusing on the standardization of the MetS definition across different research are crucial. The establishment of consistent criteria would enhance the reliability and validity of research findings, enabling more meaningful comparisons and interpretations.

## 1. Introduction

Metabolic syndrome (MetS), formerly known as insulin resistance syndrome or syndrome X, is a cluster of interrelated metabolic risk factors that increase the risk of cardiovascular morbidity and mortality [1,2,3,4,5,6]. The frequently documented risk factors include insulin resistance or glucose intolerance, central/abdominal obesity, hypertension, increased levels of low-density lipoprotein cholesterol (LDL-C), decreased high-density lipoprotein cholesterol (HDL-C), and hypertriglyceridemia (high blood triglycerides) (TG). These risk factors are major determinants of cardiovascular disease (CVD) as well [1,3,7,8,9]. Metabolic syndrome has gained significant interest worldwide due to its escalating prevalence, which places a heavy burden on the global healthcare system [10]. A remarkably high prevalence of MetS has been reported in Western communities [11], Asians [12,13,14], Africans [15], and in countries of the Middle East [16].

MetS is a product of interaction between genetic and lifestyle factors such as obesity, high-calorie diet, lack of physical activity, and smoking. However, its pathogenesis has not yet been determined [17,18,19]. The syndrome’s progression can be slowed down and even reversed with intervention to reduce the burden of MetS [20,21,22]. Hence, there is an urgent need, based on ethical, medical, and economic perspectives, to promptly identify individuals with MetS in order to intervene early. The goal is to avert the onset and progression of its associated complications, including T2DM and CVD. By identifying individuals with MetS at an early stage, interventions can be implemented in a timely manner, potentially mitigating the development and exacerbation of these significant health conditions. This proactive approach holds promise for improving overall health outcomes and reducing the burden on healthcare systems [23,24,25].

It is important to note that there is no universally agreed-upon definition of MetS. Subsequently, the prevalence of MetS varies considerably among different studies and across different populations [26]. Indeed, it is essential to note that the definitions of MetS have undergone changes and refinements over time. These changes have been driven by advancements in scientific understanding, emerging evidence, and consensus among experts in the field [27,28]. However, the precise chronology and factors influencing the development and modifications of these definitions remain less documented and require further investigation.

It is also noteworthy to highlight that the historical origins of MetS and the evolution of its definitions have not been clearly documented in the literature. The lack of a comprehensive understanding of the historical context and trajectory of MetS definitions hinders establishing standardized criteria that facilitate accurate prevalence estimations and cross-population comparisons. To address these knowledge gaps, this review aims to examine the developmental trends of MetS over the past two decades, with a particular focus on its definition, diagnostic criteria, and prevalence. Such insights can inform future research, clinical practice, and policymaking, ultimately leading to more effective prevention and management strategies for MetS.

## 2. Methods

A comprehensive literature search was conducted to examine the developmental trends of MetS, utilizing scientific databases such as Scopus, PubMed, and MDPI, covering publications in the last two decades. The search strategy incorporated relevant keywords and keyword compinations, including “Metabolic Syndrome”, “insulin resistance”, “Type 2 Diabetes mellitus”, “obesity”, “waist circumference”, and “cardiovascular disease”. Inclusion criteria encompassed studies that provided insights into the historical development, diagnostic criteria, and prevalence of MetS, while articles primarily addressing its pathophysiology, clinical features, and treatment strategies were considered when relevant to the historical discussion. Exclusion criteria involved non-English studies, duplicate articles, conference abstracts, and animal studies or studies conducted in vitro.

Titles and abstracts were screened to identify studies aligning with the scope of this review. Subsequently, full-text evaluations were conducted to extract key findings regarding the evolution of MetS definitions, changes in diagnostic criteria and their implications for reported prevalence rates. This review follows the approach of a narrative review, ensuring a rigorous synthesis of the available literature while acknowledging certain limitations, including such as the availability and accessibility of certain articles, potential publication bias, and the inherent limitations of the selected studies. However, efforts were made to include a diverse range of studies for a comprehensive review. Overall, this methodology aimed to gather and synthesize relevant information from various sources to provide a better understanding of the historical context, evolution, and prevalence of MetS.

## 3. Metabolic Syndrome: Historical Overview

The association between hyperinsulinemia, insulin resistance, hypertension, dyslipidemia, and diabetes has been thoroughly investigated over the past decades [29]. In 1920 a Swedish physician, Kylin, made a notable observation regarding the interplay between hypertension, hyperglycemia, and gout. Subsequently, he noted that visceral obesity is linked to the development of CVD and T2DM [30,31]. In 1947, Vague noted that upper-body obesity was commonly associated with metabolic abnormalities observed in patients with T2DM and CVD [27]. Over time, there has been a growing scientific interest in the topic of MetS, leading researchers to propose various theories about its underlying mechanisms [31]. Later, in 1988, Reaven described Syndrome X as a group of risk factors associated with the development of T2D and CVD in addition to the theory of insulin resistance [32].

In 1989, Kaplan retitled the syndrome “The Deadly Quartet”, adding obesity or visceral obesity as a major abnormality [33]. In 1992, the condition was renamed again as “The Insulin Resistance Syndrome” [34]. Since then, the syndrome evolved with different combinations of factors proposed to be included. However, in 1999, the World Health Organization (WHO) identified insulin resistance as the major underlying contributor [35,36]. Moreover, the diagnosis of MetS necessitated the coexistence of at least two additional risk factors, including hypertension, obesity, raised TG, or low HDL [29]. Subsequently, the coexistence of these metabolic abnormalities and the co-presence of obesity is currently referred to as metabolic syndrome [7,30,37].

It has been widely accepted that insulin resistance is almost universally present in all the conditions of MetS, although obesity seems to play a similarly important role as well [38,39]. It also has been accepted that a clustering of interrelated metabolic risk factors mentioned earlier significantly increase the risk of chronic conditions, including T2DM and CVD [2,40]. Together they account for roughly two-thirds of deaths worldwide [2,40]. Identifying people with MetS reduces the long-term risk of developing T2DM, CVD, other forms of atherosclerotic disease, obstructive sleep apnea, and nonalcoholic fatty liver disease [41,42].

Metabolic syndrome has also been referred to by several different names: pre-diabetes, insulin resistance syndrome, syndrome X, dysmetabolic syndrome, dyslipidemic hypertension, cardiometabolic syndrome, hypertriglyceridemic waist, and the Deadly Quartet [3,39]. It should be highlighted that formal definitions of the MetS have only emerged in the past 20 years. However, there is still much to uncover and understand about this syndrome [17,37].

## 4. Metabolic Syndrome: Definition

Developing a standardized definition for MetS has posed challenges, and the accurate descriptions and diagnostic criteria for MetS have undergone rapid changes in recent years. This can be attributed to the absence of a universally agreed-upon definition for MetS [43,44]. Over the years, several definitions of the MetS have been proposed, and while there is general agreement regarding the key factors associated with MetS, there have been variations among these definitions. Hyperglycemia, central obesity, atherogenic dyslipidemia, and hypertension are widely recognized as risk factors of MetS [45,46,47,48,49]. However, the emphasis placed on specific factors and the criteria for diagnosis has differed across definitions as outlined in Table 1.

For some definitions, the presence of insulin resistance has been considered a fundamental factor for diagnosing MetS. Other definitions have highlighted the significance of obesity as a primary factor. However, there are alternative definitions of MetS that do not necessarily consider obesity and insulin resistance as prerequisites for a diagnosis. These alternate definitions propose that the presence of three out of the five components mentioned earlier (central obesity, atherogenic dyslipidemia, higher glucose levels, hypertension, and decreased high-density lipoprotein cholesterol) would be sufficient to diagnose MetS. These definitions acknowledge that while obesity and insulin resistance commonly coexist with MetS and play a significant role in its development, they may not be present in all cases [4,50]. Understanding the evolution of these definitions provides valuable insights into the development of the concept of MetS. The subsequent section provides an overview of the historical progression in defining MetS.

In 1999, the WHO first published formal criteria to define the MetS in an attempt to achieve comparable reporting of prevalence through epidemiologic studies [3,45,48]. In addition to the presence of insulin resistance (i.e., impaired glucose tolerance, impaired fasting glucose, type II diabetes mellitus, or lowered insulin sensitivity), there must be two or more of any other metabolic risk factors for the diagnosis of MetS. The latter are central obesity as WHpR > 0.90 for men or >0.85 for women and/or BMI > 30 kg/m^2^, dyslipidemia as TG ≥ 150 mg/dL and/or HDL < 35 mg/dL in men or <39 mg/dL in women, blood pressure ≥ 140/90 mmHg, and microalbuminuria ≥ 20 µg min^−1^ or albumin: creatinine ratio ≥ 30 mg g^−1^. The WHO has also described several other components of the MetS (e.g., hyperuricemia, coagulation disorders, raised plasminogen activator inhibitor (PAI)-1, etc.), but they were not considered mandatory for the diagnosis of the syndrome [37]. Since measurements of microalbuminuria and insulin resistance (using a euglycemic clamp) are laborious and cannot be easily used in both clinical practice and epidemiological studies, several alternative definitions have been proposed [27].

Later in 1999, the European Group for the Study of Insulin Resistance (EGIR) released a revised version of the WHO definition [51]. EGIR identified specific challenges associated with the application of the WHO criteria. First, the use of the euglycemic clamp method to measure insulin resistance was deemed impractical in field settings. Second, the evidence supporting a strong correlation between microalbuminuria and insulin resistance was found to be weak. Lastly, measuring waist circumference was considered more convenient than assessing the waist-to-hip ratio, as the former exhibited a closer correlation with obesity. These insights prompted EGIR to modify the WHO definition, addressing the limitations and enhancing the overall understanding of MetS [49,51].

The EGIR definition focused more on abdominal obesity and introduced waist circumference (94 cm for men and 80 cm for women) as the measure of obesity [52]. The proposed EGIR definition excluded subjects with diabetes because of difficulties in measuring insulin resistance in these individuals as beta-cell dysfunction, a key characteristic of T2DM, makes estimates of insulin sensitivity unreliable. However, insulin resistance remained an essential component, defined as a fasting insulin level above the 75th percentile for the population. Hence, it was renamed as insulin resistance syndrome [43,52].

In 2001, The National Cholesterol Education Program (NCEP) and Adult Treatment Panel III (ATP III) published a new definition of the MetS, focusing on CVD risk factors, relying less on measures of insulin resistance as criteria [17,52]. They identified the following components of MetS as part of CVD risk factors: abdominal obesity, atherogenic dyslipidemia, raised blood pressure, and insulin resistance/glucose intolerance [53]. Thus, NCEP: ATP III adopted three or more of five metabolic risk factors: central obesity as WC ≥ 102 cm for men or ≥88 cm for women, dyslipidemia as TG ≥ 150 mg/dL, and HDL < 40 mg/dL in men or <50 mg/dL in women, blood pressure ≥ 130/85 mmHg, and fasting blood glucose ≥ 110 mg/dL [37]. The latter value was modified to be 100 mg/dL according to the American Diabetes Association’s updated definition of impaired fasting glucose [37]. The NCEP: ATP III definition also employed waist circumference as the measure of obesity because it is believed to be a good index for the identification of central obesity and obesity-associated risk factors [10,41], but with higher cut-off points than EGIR (102 cm for men and 88 cm for women). In comparison to the WHO definition of the MetS, the ATPIII definition components are easily and routinely measured in most clinical and research settings. Subsequently, it has been commonly used due to its simplicity [27].

In 2003, the American Association of Clinical Endocrinologists (AACE) modified the ATP III criteria and renamed the disorder “Insulin Resistance Syndrome” due to their belief that insulin resistance was at the heart of the syndrome [52]. The AACE criteria to define the syndrome were flexible, describing four vital metabolic abnormalities including insulin resistance, elevated triglycerides, reduced HDL-C, and elevated blood pressure [54]. The AACE used the NCEP: ATP III criteria of dyslipidemia and hypertension in their definition [54]. Diabetic patients were excluded from the definition criteria [13]. Other abnormalities include markers of inflammation, abnormalities in uric acid metabolism, hemodynamic changes, prothrombotic factors, and endothelial dysfunction [31]. Obesity was excluded as a component of the MetS as the AACE considered central obesity a causative factor in developing insulin resistance rather than as a consequence [13]. This has resulted in much disapproval as obesity is widely accepted as a major risk factor for T2DM and CVD [55].

In 2005, the International Diabetes Federation (IDF) set out a task force to better define the nature of the MetS and to produce a new set of criteria for use both epidemiologically and in clinical practice worldwide. This was an important step towards identifying people with the syndrome. The new definition makes central obesity a mandatory requirement, plus two or more of any other metabolic risk factors of NCEP: ATP III [27,30,56,57]. An important feature of the IDF criteria is the development for the first time of ethnic-based cut-off points to compensate for differences in waist circumference and fat distribution among diverse populations [27]. The WC is based on population estimates: European ≥ 94 cm for men and ≥80 cm for women; South Asian, Chinese, and Japanese ≥ 90 cm for men and ≥80 cm for women. For other populations, the IDF recommended that Ethnic South and Central Americans use South Asian recommendations until more specific data are available and that Sub-Saharan Africans, Eastern Mediterranean, and the Middle East (Arab) should use European data until more specific data are available [27,56].

The variation in the WC cut-off points was attributed to ethnic differences in body fat content that are higher in Asians than Caucasians [58]. For instance, it should be taken into consideration that Asians have a higher upper-body fat than Caucasians who have a higher lower-body fat [41]. Africans also have a different body composition; they have a higher percentage of bone and muscle than Caucasians [59]. Studies in the Arab world have yielded inconsistence estimates for the WC cut-off points, emphasizing the need for further research in the region [60]. Taking into account ethnic differences in body fat content and distribution is likely to enhance the identification of individuals with the syndrome both globally and in clinical practice. The development of new criteria for diagnosing MetS is ongoing and standardizing proper definition of MetS is important. Indeed, standardizing the definition of MetS across studies could enhance the comparison of its prevalence and impact among different populations [16].

## 5. Metabolic Syndrome: Prevalence

Metabolic Syndrome is a widespread disorder worldwide, with a prevalence ranging from 14–39% in different countries [25,43,61,62]. Due to the increased consumption of Western-style diets, and sedentary lifestyle, globally, MetS is reaching epidemic levels [63,64]. As a consequence, levels of T2DM and CVD is also likely to rise [65]. Several factors affect the differences in the prevalence of MetS among populations. This include the differences in ethnicity, age, sex, geographic location as well as the criteria used for the definition of MetS [13,61,66]. The prevalence of MetS increases with the age group for both sexes [12,67]. The overall prevalence of MetS in the United States (US) is higher in people over 60 years with rates of 54% compared to 20% in those over the age of 20 years [68].

Regarding gender, women aged 60 years and older exhibit a greater likelihood of meeting the criteria set by NCEP: ATP III [67,68]. Conversely, this association appears to be less pronounced among younger age groups of women [67,68]. The prevalence of MetS was the highest in white men (35%), and lowest in African American men (21.6%) [69]. Mexican American women had the highest overall prevalence of 37.8%, whereas African American women and white women had almost similar prevalence of (25.7% and 22.8%) respectively [61,69]. Lower prevalence rates of MetS were observed among rural populations compared to urban populations [13,70,71]. Furthermore, a higher prevalence was observed among Arabs living in the United States (consuming a Western-style diet) compared to those living in the Middle East [13].

Several studies suggest that using the IDF criteria would lead to a higher prevalence of MetS compared to the ATPIII criteria. This is explained by the fact that lower cut-off points for waist circumference is used by the IDF definition [7,61,72]. According to the National Health and Nutrition Examination Survey (NHANES) data gathered from 1999 to 2002, approximately 34.6% of the United States population met the criteria for metabolic syndrome when evaluated using the ATP III framework; however, this prevalence rose to 39.1% when the IDF criteria were employed instead [61].

Later, NHANES reports during the period 2011–2016 showed an elevation in the prevalence of MetS that reached 39.38% (35.97–42.79%) in men and 36.11% (33.32–38.90%) in women of the Asian American Adult population using the ATP III criteria. The overall prevalence estimate increased to 39.26% (35.91–42.60%) in men and 39.66% (36.93–42.39%) in women using the IDF criteria [73].

The prevalence of metabolic syndrome (MetS) differs across nations, as evidenced by various studies [74,75]. In the United States, the age-adjusted prevalence among adults stands at 34.3% [76], whereas in Sweden, it is recorded at 14.8% for men and slightly higher at 15.3% for women. In contrast, Italy reports a prevalence of 19.6% among men, yet the figure for women is notably elevated, reaching 33.3%. Meanwhile, in India, research indicates that 13% of the sampled population exhibits MetS [77], and within Arab countries, the prevalence fluctuates between 17% and 34.6%, depending on the specific region [78,79].

The levels of obesity, a major component of MetS, range from under 5% in China and Japan to over 75% in urban Samoa [80]. Based on data extracted from the NHANES Survey, there has been a notable increase in the age-adjusted prevalence of obesity in the United States. Specifically, the prevalence increased from 22.9% during the period of 1988–1994 to 30.5% in 1999–2000. Subsequently, there was a further rise in obesity prevalence, reaching 35% during the period of 2011–2014 [81].

The healthcare costs are much higher if the obesity-related complications of T2DM and CVD are also considered [82]. The prevalence of T2DM is rising due to the exponential increase of MetS [39]. The prevalence of diabetes in all age groups worldwide was estimated to be 2.8% in 2000, and it was projected to become 4.4% in 2030 [83]. However, T2DM now affects approximately 6.28% of the world’s adult population in developing countries, with the highest rates in the Eastern Mediterranean and Middle East regions [84]. The prevalence of CVD (another sequel of MetS and a major cause of morbidity and mortality) is also increasing, estimated at 48% in the US in 2016; resulting in a huge economic burden on the healthcare system [7,85].

It is noteworthy to mention that sex differences in the prevalence of metabolic syndrome are significant and vary based on factors such as age, ethnicity, and lifestyle [86]. Previous research showed that men exhibit a higher prevalence of metabolic syndrome than women [87]. This might be due to the fact that men tend to have higher rates of abdominal obesity and lower HDL cholesterol levels [88]. However, women often show an increase in prevalence post-menopause, largely due to hormonal changes. Women may present with elevated blood pressure and triglyceride levels, particularly after menopause [89]. However, lifestyle factors, including sedentary behavior and poor dietary habits, further contribute to the development of metabolic syndrome, with differences in lifestyle choices between genders impacting these rates [90]. Understanding these differences is essential for developing targeted prevention and treatment strategies for MetS.

## 6. Pediatric Metabolic Syndrome

Pediatric MetS has emerged as a major public health concern. In 2020, it was estimated that approximately 25.8 million children and 35.5 million adolescents were affected by MetS [91]. It is important to acknowledge that a significant number of health issues that manifest in adulthood are often linked to factors developed during childhood. For instance, children with MetS are 2.4 times more likely to develop MetS as adults and 2.6 times more likely to experience T2DM in adulthood compared to those without these conditions [92]. Furthermore, children with MetS are 14.7 times more likely to develop cardiovascular disease in adulthood compared to healthy children [93]. Therefore, it is important to consider strategies aimed at reducing MetS in children to help prevent chronic diseases in adulthood.

It is noteworthy to mention that the significant occurrence of pediatric MetS corresponds with the increasing childhood obesity rates [94]. While the prevalence of MetS among children and adolescents is reported to range between 2.8% and 4.8%, it is notably higher in the obese population [91]. For instance, a recent systematic review and meta-analysis published in 2025 analyzed 57 studies with 27,923 participants and found that the average prevalence of MetS in children and adolescents with obesity was 26% (95% CI: 0.22–0.30; I^2^ = 98%). This suggests that, approximately, 1 in 4 children with obesity is affected by MetS [95]. Childhood obesity increases the likelihood of developing MetS through several interconnected mechanisms. Firstly, excess body fat, particularly visceral fat, leads to insulin resistance, where the body’s cells become less responsive to insulin, resulting in elevated blood glucose levels. Secondly, obesity is associated with dyslipidemia, characterized by high triglycerides and low levels of HDL cholesterol, which are significant risk factors for MetS and cardiovascular diseases. Additionally, obese children often experience chronic low-grade inflammation due to the secretion of inflammatory cytokines from excess adipose tissue, impairing insulin signaling and promoting metabolic dysfunction. Furthermore, childhood obesity frequently leads to a clustering of metabolic risk factors, such as hypertension and impaired glucose tolerance, which significantly increases the likelihood of developing MetS [96].

Hence, identifying and addressing MetS in children and adolescents at an early stage are tasks of considerable importance. However, similar challenges arise in defining MetS for children and adolescents as those encountered in adults. Specifically, there is considerable variability in the definitions of pediatric MetS among, with approximately 40 distinct definitions currently in use [97]. The lack of a standardized definition has made it challenging for healthcare professionals to assess and address MetS effectively in pediatric populations [98]. Therefore, in 2024, Zong and colleagues proposed a simplified definition of MetS to facilitate direct comparisons of its prevalence across diverse pediatric populations.

The proposed simplified definition of MetS for children and adolescents aged 6 to 17 years is derived from two established definitions: the IDF and NCEP criteria. This definition uses fixed cut-off values for its five components: central obesity, high BP, elevated TG, low HDL-C, and high fasting blood glucose (FBG). To meet the criteria for MetS, at least three of these components must be present, with central obesity not being deemed a mandatory component [99]. Central obesity is identified using waist-to-height ratio thresholds of ≥0.50 for youths in Europe and the USA, and ≥0.46 for those in Asia, Africa, and South America. High BP is defined as ≥130/80 mm Hg for adolescents aged 13–17 years and ≥120/80 mm Hg for children aged 6–12 years. Elevated triglycerides are classified as ≥130 mg/dL for those aged 10–17 years and ≥100 mg/dL for ages 6–9, while low HDL-C is recognized as <40 mg/dL. Lastly, high FBG is set at ≥100 mg/dL. This new definition aims to enhance the consistency of monitoring MetS risk among children and adolescents, ultimately aiding in the development of evidence-based prevention strategies on a global scale [99].

Further research is needed to validate this simplified definition and its applicability across diverse populations. Longitudinal studies should be conducted to assess the effectiveness of this definition in predicting long-term health outcomes related to MetS. Understanding the role of genetics, lifestyle choices, and dietary habits will also be essential in creating comprehensive prevention strategies. Moreover, it is recommended to establish standardized screening protocols that can be easily implemented in clinical settings to facilitate early detection of MetS in pediatric populations.

## 7. Metabolic Syndrome: Pathophysiology

**Figure 1:**
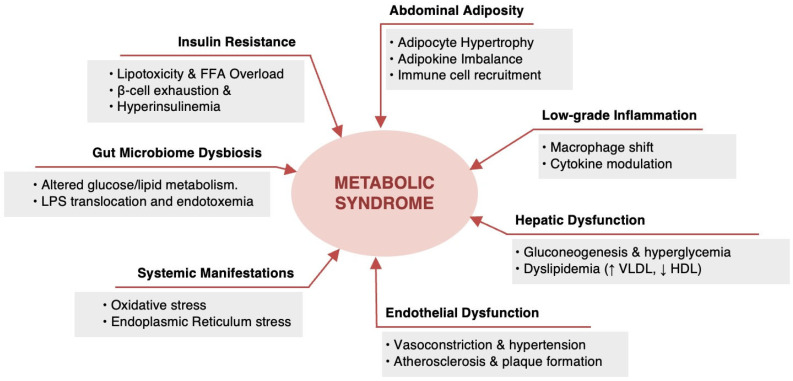
Pathophysiological mechanisms of metabolic syndrome.

The pathophysiology of MetS is controversial and is not yet completely determined but it is mainly attributed to insulin resistance and central obesity [37]. The relationship between insulin resistance and central obesity is complex [100]. Both of them lead to subsequent metabolic risk factors, particularly hyperglycemia, dyslipidemia, and hypertension that predispose to T2DM and CVD [30,101]. Other contributory factors include genetic or ethnic predisposition, aging, physical inactivity, high caloric intake, cigarette smoking, Western-style diet, pro-inflammatory state, and hormonal imbalance [101,102]. A sophisticated factor analysis study by Akter, et al. identified three key factors for the development of MetS [103]. These factors are (1) a “metabolic” factor (including WC, BMI, TG, IGT test, insulin sensitivity, and PAI); (2) an “inflammation” factor (including WC, BMI, C-reactive protein (CRP), fibrinogen, and insulin sensitivity); and (3) a “blood pressure” factor [103]. However, many clinical features of the syndrome as well as the mechanisms of MetS development are still poorly understood, highlighting the need for further research to clearly understand the pathophysiology of the syndrome. Figure 1 illustrates the possible pathophysiological mechanisms of MetS.

The complex pathophysiology of MetS stems from the interactions between the metabolic and inflammatory pathways. While insulin resistance and central obesity are widely recognized as core contributors, emerging evidence highlights the role of chronic low-grade inflammation as a key underlying mechanism driving MetS progression [104]. Adipose tissue dysfunction, particularly in visceral fat depots, leads to excessive secretion of pro-inflammatory cytokines such as tumor necrosis factor-alpha, interleukin-6, and CRP. These inflammatory mediators disrupt insulin signaling pathways, exacerbate endothelial dysfunction, and contribute to hypertension, dyslipidemia, and hyperglycemia [105,106]. Moreover, emerging evidence suggests that metabolic endotoxemia, characterized by increased circulating lipopolysaccharides due to gut microbiota dysbiosis, serves as an additional driver of inflammation in MetS. Lipopolysaccharides activate toll-like receptor 4 signaling, further promoting cytokine release and extending metabolic pathway dysfunction [107]. Eventually, the interplay between insulin resistance, central obesity, and chronic low-grade inflammation emphasizes the multifactorial nature of MetS, demanding further research to explain its precise mechanisms and develop targeted interventions for its prevention and management.

In summary, the prevalence of MetS is a significant global health concern, with rates ranging from 14% to 39% across different countries. However, it is important to note that the prevalence of MetS varies considerably according to the different definitions. Additionally, it differs within and across diverse populations, ethnicity, sex, and age group. Nonetheless, sedentary lifestyles and Western-style diets contribute to the increasing epidemic of MetS. The rising prevalence of MetS contributes to the increasing rates of T2DM and CVD, leading to significant healthcare costs and economic burdens. Efforts to address the risk factors and raise awareness about prevention strategies are crucial in mitigating the impact of MetS on public health.

## 8. Conclusions

In conclusion, MetS is a complex and multifaceted condition characterized by the interplay of various metabolic risk factors, including but not limited to central obesity, insulin resistance, dyslipidemia, and hypertension. There is a variation in its prevalence across different populations and sociodemographic characteristics. This could be attributed to the lack of precise definition criteria among these different populations. Hence, it is imperative to standardize the definition of MetS across studies. Standardization would enable researchers to employ a consistent set of criteria, enhancing the reliability and validity of findings. The historical evolution of MetS definitions reflects an ongoing endeavor to capture the intricate nature of this syndrome and provide a standardized framework for its diagnosis and management. The multifactorial nature of MetS is driven by a combination of genetic, lifestyle, and environmental factors. While insulin resistance and central obesity have been traditionally considered central to its pathophysiology, emerging evidence underscores the significant role of chronic low-grade inflammation in the development and progression of MetS. Inflammatory cytokines, oxidative stress, and gut microbiota alterations drive the inflammatory state and contribute to metabolic dysfunction, suggesting that future research should explore novel therapeutic strategies targeting these pathways. An expanded understanding of the underlying factors associated with MetS, along with the refinement of diagnostic criteria, and the implementation of lifestyle modifications and early interventions, as well as a clearer understanding of the interrelationship between metabolic and inflammatory pathways, holds the potential to yield substantial advancements in mitigating the public health impact of this condition. However, further research and collaboration are needed to improve the diagnosis, management, and prevention of MetS.

## Figures and Tables

**Table 1:** Summary of the major metabolic syndrome definitions.

MetS Component	WHO, 1999	NCEP: ATPIII, 2001	IDF, 2005	IDF and AHA/NHLBI, 2009
Obesity	BMI > 30 kg/m^2^ and/or WHpR > 0.9 in men, >0.85 in women	WC ≥ 102 cm for men and ≥88 cm for women	WC ≥ 94 cm for European men and ≥80 cm for European women, with specific values for other ethnic groups	Increased WC (Population- and country-specific cut-off points)
Hypertension	blood pressure ≥ 140/90 mmHg or on antihypertensive treatment	blood pressure ≥ 130/85 mmHg	blood pressure ≥ 130/85 mmHg or treatment of previously diagnosed hypertension	blood pressure ≥ 130/85 mmHg or treatment of previously diagnosed hypertension
Hypertriglyceridemia	≥1.7 mmol/L	≥1.7 mmol/L	≥1.7 mmol/L or specific treatment for this lipid abnormality	≥1.7 mmol/L or specific treatment for this lipid abnormality
Low HDL-C	<0.9 mmol/L in men or <1.0 mmol/L in women	<1.04 mmol/L in men or <1.29 mmol/L in women	<1.03 mmol/L in men or <1.29 mmol/L in women or specific treatment for this lipid abnormality	<1.0 mmol/L in men or <1.3 mmol/L in women or specific treatment for this lipid abnormality
Hyperglycemia	IR, identified by one of the following: T2DM, IFG ≥ 6.1 mmol/L, IGT ≥ 7.8 mmol/L, Hyperinsulinemia, euglycemic conditions with low glucose uptake	IFG ≥ 6.1 mmol/L	FBG ≥ 5.6 mmol/L or previously diagnosed T2DM	FBG ≥ 5.6 mmol/L or previously diagnosed T2DM
Others	Microalbuminuria: Urinary albumin excretion rate ≥ 20 µg/min or albumin/creatinine ratio ≥ 30 mg/g			
MetS criteria	IR PLUS any TWO other components	three or more needed	Obesity PLUS any TWO other components	three or more needed

Abbreviation; Mets: Metabolic Syndrome; WHO: World Health Organization; NCEP: ATPIII: National Cholesterol Education Program: Adult Treatment Panel III; IDF: International Diabetes Federation; AHA/NHLBI: American Heart Association/National Heart: Lung: and Blood Inst.
